# Repertory of the Back

**Published:** 1898-10

**Authors:** E. H. Wilsey

**Affiliations:** Parkersburg, W. Va.


### REPERTORY OF THE BACK.

E. H. Wilsey, M. D., Parkersburg, W. Va.

(Continued from September number, page 413.)

### SCAPULÆ.

**Between**, gnawing between scapulæ, Alum, Nat-carb.
— heat between scapulæ and sternum, Arg-nit.
— heat between scapulæ, *Phos.*
— ice, as if a piece of ice were lying on back between scapulæ, and followed by a chill with goose-flesh all over, Lachnan.
— itching on and between scapulæ, **Alumina.**
— itching between scapulæ in evening with much eruption, Zinc.
— itching and tickling stitches between scapulæ, Arg-m.
— itching and pain between scapulæ, Gambog.
— jerking, pressing between scapulæ, extending through to epigastrium while sitting, *Bry.*
— jerking from posterior walls of chest to between scapulæ synchronous with the heart beat and anxiety, Calc-ac.
— jerking pain between scapulæ and in region of left pelvis during rest with painful jerking on moving, Sul.
— lancinating pain between scapulæ, Agari.
— lancinating between scapulæ, in shoulders and along spine to sacrum, worse by straightening himself, Ginseng.
— lancinating pain between scapulæ so that he had to bend forward while walking, Colocy.
— lancinating pain between scapulæ with oppression and anguish on chest almost solely while sitting, so that he must rise and walk, Kali-c.
— load, feeling as if a heavy load was hanging on back between scapulæ, Carbo-s.
— menses, pain between scapulæ with suppressed menses, *Ars.*
— mornings, pain between scapulæ mornings on awaking, Æthusa.
— mornings, pain between scapulæ mornings, Podo.

SCAPULÆ.

**Between,** moving, tensive pain between scapulæ when lying down or moving, Sul.

— neuralgic pain between scapulæ and in neck, Tabac.

— pain between scapulæ and in neck, *Eupat-perf.*

— pain between scapulæ, Amm-c., Canth., Calc-phos., Cup., Curare, Fer., Hepar., *Nitr-ac.*, *Ox-ac.*, Phos., Ran-scl., Tell., Therid., Medor.

— pain between scapulæ by moving arms, Naja.

— pain between scapulæ and in lower part of the back, Calc-c.

— pain between scapulæ rending asunder, Sil.

— pain between scapulæ and a little lower down the back, Calabar.

— pain between scapulæ on chest., Lach.

— pain between scapulæ and down back constantly, Sepia.

— pain between scapulæ and in lumbar region and small of back, Kobalt.

— pain between scapulæ running to either side, shoulder, and down to lumbar region, Medor.

— pain between shoulders extending to loins from under point of scapulæ, Ox-ac.

— paralytic pain between scapulæ, and in nape mornings, Nat-c.

— piercing as from knives between scapulæ when sitting in evening, Nat-s.

— pimples between scapulæ, large with burning sensation, Lyco.

— pinching between scapulæ as with pincers, Nit-acid.

— pinching between scapulæ, *Ipec.*, Nit-acid.

— pinching constriction between scapulæ, *Calc.*

— pinching between scapulæ, with coldness, Viola-tri.

— pressing pain between scapulæ, which seem to extend from posterior wall of stomach, Arn.

— pressing pain between scapulæ, when stepping hard or on other movements which concuss the chest, Senega.

— pressing pain between scapulæ, with a pressing under right scapulæ, Lyss.

— pressive drawing between scapulæ, Calend.

— pressive pain in spine between scapulæ with short breath,

SCAPULÆ.

worse in respiring, with pain in spinal vertebræ on being touched, Calc-c.

**Between**, pressure between scapulæ, Æthusa., Arum-t., Ars-hy., Graph.

— pressure dull between scapulæ, Lob-i.

— pressure between left scapulæ and spine, Led.

— pressure painful between scapulæ, Calend., Lyss., Petrol.

— pressure between and under both scapulæ, *Calc-carb.*

— pressure inward between scapulæ, Lauroc.

— pressure between scapulæ which on motion impedes respiration, beneath right scapulæ extending upward, *Calc-carb.*

— pressure between scapulæ on awaking, Thuja.

— pressure strong on a small spot of upper part of scapulæ Sepia.

— pressure pain in back between scapulæ, as if parts had been strained or had suffered injury with a like pain in front part of chest on moving, Carbo-an.

— scapulæ, pressure as from a sprain and pressure extending forward into chest, Pet.

— pressure and bruised pain in scapulæ, *Graph.*

— pulsating between scapulæ, a pain passing from one to the other, thence to dorsum of right arm in extensor muscle darting toward elbow, Plant.

— pulsating stitch in the os coccyx when sitting, also between scapulæ, Paris-q.

— raising, pain between scapulæ and in lower part of chest and in left scapula, worse by raising arm, Clem.

— respiration, sticking between scapulæ cutting short respiration, Copaiva.

— rheumatic pressure between scapulæ, worse by moving arms, when walking about 4 A. M., Sil.

— rheumatic, violent rheumatic pain neither better nor worse by motion or rest, only better by warmth and worse by cold, Rhus.

— rheumatic pain between scapulæ, Aspar., *Camph.*, Lob-i., Mag-s., Rhod.

SCAPULÆ.

**Between,** rheumatic pains in back and between inferior angles of scapulæ, Bry.

— rheumatic pains between scapulæ, can hardly turn in bed, feels beating of pulse when lying, Calad.

— rheumatic pains between scapulæ mornings on waking, Ran-b.

— rheumatism between scapulæ extending from neck to small of back, Ver-a.

— rheumatism between scapulæ on moving extending to sacrum, Drosera.

— sacrum, pain between scapulæ extending to sacrum, Zinc.

— a scraping between scapulæ, Nux-m.

— sensitive spot between scapulæ, when touched he has water-brash followed by nausea and vomiting, *Rhus.*

— sensitive spinous process of dorsal vertebræ between scapulæ become very sensitive to touch, much worse on left side, *Phos.*

— severe pain between scapulæ; he inclines forward for relief; worse from inspiration, it passes gradually around in front to ninth and tenth ribs, Nat-ars.

— shocks upward to between scapulæ from posterior walls of chest, *Calc-c.*

— shooting between scapulæ, has to straighten up, Bov.

— shooting between scapulæ extending up nape of neck, then soreness of occipital region with disposition to throw the head back, Polyporus.

— shooting pressure between scapulæ, Sepia.

— shooting and other pains between shoulders and along borders of scapulæ, has to straighten up to be relieved, Bov.

— sore between lower angles of scapulæ, afterward it extended up and down the spine, Convall.

— soreness between scapulæ with dry cough, two short hacks, Sul-acid.

— smarting between scapulæ, *Kali-b.*

— sprain, pain between scapulæ as from a sprain and pressure extending forward into the chest, Petrol.

SCAPULÆ.

**Between**, sprained pain between scapulæ and forepart of chest on moving arms, Carbo-an.

— sprained, in muscles between left shoulder and neck as if sprained on rising from sleep at 4 P. M., Chin-ars.

— a sudden sprained pain between scapulæ on lifting a weight, worse in left with sticking upon slightest movement, breathing or yawning, and on bending backward intolerable pain, Stann.

— squeezing, stitches between scapulæ, worse on right side, *Cepa.*

— sticking between scapulæ, *Agari.*, Colchi., Dig., Hepar., *Lach.*, Lyco., **Mang.**, Millef., Psori., Thuja, Sepia., Sarsa.

— sticking fine between scapulæ and in back, *Puls.*

— sticking between scapulæ on deep inspiration, Nat-ars.

— sticking between scapulæ in region of third dorsal vertebræ in morning, Ran-b.

— sticking intermittent between scapulæ, Kreos.

— sticking extending outward in spine between scapulæ, Stann.

— sticking between scapulæ, relieved by walking, Nat-c.

— sticking between scapulæ in evening with tearing, better in daytime, returning at night in warmth of bed, Sul.

— sticking between scapulæ after dinner, Phelland.

— stiffness between scapulæ, Caust., *Kali-c.*, Lact-acid.

— stiffness between scapulæ painful, Ang.

— stiffness, pain between scapulæ with stiffness from neck down to between scapulæ, *Apis.*

— stiffness between scapulæ extending to neck, Agari.

— stinging between scapulæ, Alum.

— stinging, transitory stitches between scapulæ, Asclep-tub.

— stitch between scapulæ, Indigo., Mang.

— stitch occasionally between scapulæ, always succeeded by eructation, Nit-a.

— stitches between scapulæ, Alumina., Ascl-t., Carbo-an., Colch., Hepar., Lyco., Paris, Plumb., Psori., Sepia., Sil.

— stitches in and between scapulæ, *Calc.*, *Cocc-c.*, *Coccul.*, *Nit-a.*, *Ran-b.*

SCAPULÆ.

**Between**, stitches between scapulæ on drawing a long breath, Aco., Pru-sp.

— stitches between scapulæ and in lumbar region, then pressure, Æthusa.

— stitches, dull between scapulæ, near spine, Ang.

— stitches between scapulæ as if sprained on every motion, Canth.

— stitches in and between scapulæ with arrest of respiration, Alum.

— stitches, dull, between scapulæ extending from behind forward in afternoon while lying down, Bry.

— stitches between scapulæ in evening when sitting, Nat-sul., *Bry.*

— stitches, slow, intermitting, dull, between scapulæ in middle toward spine, Stann.

— stitches, violent, small, between scapulæ, Sarsa.

— stitches, between scapulæ and anteriorly in the chest, arresting the breath more when stooping than when sitting still, Nit-a.

— stitches, obtuse, between scapulæ, Con., Dig.

— stitches between scapulæ obstructing breathing at night, Carbo-veg.

— stitches, severe, from the thoracic cavity, through spine out between scapulæ, Calc-c.

— stitches, broad, sharp, in the spine, between scapulæ, from within outward, Stann.

— stitches and tension between scapulæ, *Colch.*

— stone, pressure as from a stone between scapulæ, *China.*

— a strained feeling between scapulæ, Bell.

— a straining while stooping in the neck and between scapulæ, Ant-cr.

— tearing in and between scapulæ extending to occiput, Berb.

— tearing pain between scapulæ, Anac.

— tearing, a violent tearing pain between scapulæ, *Sil.*

— tearing frequently between scapulæ, Agaric.

— tearing between scapulæ, Anac., Berb., Calc., Calend, Canth., Mag-m., Caust., Meny., Psori., *Puls.*, *Sil.*

SCAPULÆ.

**Between**, tearing between scalpulæ so he could not move, Petrol.
— tearing between scapulæ and at same time they were drawn together, Rhus.
— tearing, painful between scapulæ, Anac., Caust.
— tearing between scapulæ and also shooting in evening, Sul.
— tearing, with feeling as if a piece would be torn off on inner border of left scapulæ deep in bone, as with a knife between scapulæ, in afternoon, Bov.
— temples, pain between scapulæ, extending over head into temples, Kalmia.
— tension painful between scapulæ; better by rubbing, Carbo-an.
— tension painful between scapulæ, *Rhus*, Sepia.
— tension between scapulæ, Nat-c., Sepia.
— tension between scapulæ in back, extending into neck, Lauroc.
— tension between scapulæ, with cutting and tingling in skin, Jacea.
— tension between scapulæ and in sternum, preventing raising arms, a kind of paralysis, Fer.
— tension between scapulæ and down back, Mag-mur.
— tension and drawing between scapulæ in open air after taking off coat, Nat-carb.
— tensive pain between scapulæ, Sepia.
— tensive aching between scapulæ and in side of neck, Sul.
— throbbing pain between scapulæ, Merc-i-fl.
— throbbing and aching pain between scapulæ, Calc-phos.
— throbbing between scapulæ, *Phos.*, Kali-iod.
— throbbing with tearing alternating, left scapulæ, then between scapulæ, also at night, Baryta-carb.
— tickling, itching stitches between scapulæ, Arg-m.
— tugging pain between scapulæ; when sitting or on turning it becomes a tugging, Ver-alb.
— twitching in cervical vertebræ between scapulæ, Phell.
— thrusts dull from posterior walls of thoracic cavity to between scapulæ in the rhythm of the heart-beat, with great anguish, Calc.
— weakness between and below scapulæ, Sarrac.

SCAPULÆ.

**Between,** weakness, a peculiar weakness and stiffness between scapulæ, extending to neck, Agari.

— wind; sensation of wind blowing on parts between scapulæ, Caustic.

— yawning, a sudden sprained pain between scapulæ on lifting a weight; worse in left side with sticking upon slightest movement; breathing or yawning or bending backward intolerable, Stannum.

**Blows**, a stinging in right scalpulæ; paining when in motion as from blows and bruises, Kali-c.

**Bones**, tearing in bones of scapulæ, Merc-c.

— beating and tearing in the right scapula, seemingly in the bone, soon recurring after friction, Phos.

— pain as if in the bone of the right scapula, Cund.

— pain in bones of right scapula with darting from right clavicle to right scapula, Ver-alb.

**Boring** pain in scapula, Cornus.

— in tip of left scapula felt as far as the end of the ensiform cartilage, Nat-carb.

— in left scapula, Aur-m-nat., Spig.

— in left scapula relieved by motion, Peonia.

— in middle of right scapula, as if extending to the ensiform cartilage, Nat-carb.

**Breath,** severe shooting in left shoulder arresting the breath, Graph.

— sticking from right scapula down side of spine to last rib, worse by inspiration, with tearing, and on deep inspiration arrest of breath, Sarsa.

— can't take a full breath for the pain under lower angle of left scapula, worse from motion or breathing, Cup-ars.

— drawing a long breath aggravates the pain under right scapula, Jug-cin.

— scapulæ seem to him swollen and the pain took away his breath if he leaned with back against anything, Sil.

— drawing and tearing in right scapula, it compels him to take a deep breath, Nat-m.

SCAPULÆ.

**Breathing,** stitches in right scapula on breathing, *Kali-C.*

— several stitches under scapula arresting breathing and not allowing any stooping, Sul.

— terrible pain from under left scapula around to chest and down to groin, constricting breathing, *Mag-phos.*

— extremely painful between scapula and down back, especially on deep breathing, Meny.

**Broken,** pain as if scapula were broken, Ananther.

**Bruised** pain in the scapula, Graph., Thuja.

— pain beneath scapula (left), Ledum.

— pain in scapula with pressure between, *Graph.*

— pain in right scapula and upper arm, worse by lifting, Anac.

— severe pain as if bruised over the entire scapular region, Merc-i-fl.

— paralytic pain in scapula as if bruised when sitting or standing, better by lying down, Asarum.

— feeling in right scapula toward back, *Arn.*, *Cicuta.*

— a bruised pain in scapula and in hips, Nat-m.

— pain as if swollen or bruised from right scapula to shoulder-joint, Berb.

— pain as if bruised between scapula, worse by stooping and in intercostal muscles, Cham.

**Bubbling** sensation beneath scapula in back and upper arm, Squilla.

— sensation in left scapula as if blood were dropping through a valve, Spig.

— on border of right scapula, near axilla, as if air was forced into flesh, Berb.

**Burning** in scapula, *Carbo-veg.*, *Rob.*, Zinc.

— about hips and in scapula, with aching along spine, Carbo-veg.

— and tensive aching between scapula, *Sul.*

— from pimples below right scapula, Cist.

— stitches below scapula while sitting, Oleand.

— violent burning below right scapula, caused by a very much inflamed spot about the size of palm of hand; soon after pimples appear on this spot in a large cluster, causing vio-

SCAPULÆ.

lent burning; later, rheumatoid pains into left hip, worse from motion, Cist-c.

**Burning** and itching on a spot below and to the right of scapula, Pallad.

— pain between scapulæ, *Phos.*

— pain often over a small space in scapulæ, greatly worse by long-continued needlework or writing, Ran-b.

— pain, then burning on right scapula, Lyco.

— pain on left scapula, Sil.

— stitches in right scapula, running to elbow, with burning pain, Sil.

— beneath scapulæ, Tabacum.

— beneath right scapula, Can-sat.

— beneath right scapula, close to spine, with heaviness in right side of chest, Staph.

— between scapulæ, *Glon.*

— between scapulæ, in myelitis, *Sul.*

— between scapulæ, before vomiting, *Rhus.*

— between scapulæ, from small of back, Thuja.

— sensation between scapulæ, with hot feeling down back, Glon., Lyco.

— and tingling between scapulæ, Saba.

— between scapulæ, when sitting, Thuja.

— in scapulæ, with tension between and extreme restlessness of extremities, *Zinc.*

— and heat in dorsal region, mostly below lower half of scapulæ, while sitting reading at night, Helon.

— and soreness between scapulæ, Phos-ac.

— and throbbing between scapulæ, *Phos.*

— pain between scapulæ, *Phos.*, Sul.

— as from hot coals between scapulæ, *Lycopod.*

— coals, burning as from red-hot coals on shoulders, Phos-ac.

— in left side and left scapula, Zinc.

— in middle of right scapula, Caustic.

— in right scapula, also in region of left kidney, Lachn.

— on right scapula, Iod.

SCAPULÆ.

**Burning** and shooting on left shoulder, extending to hip, Mag-m.
— over both scapulæ, Lachn., Sil., Sul.
— over left scapula, Ambra., Bary-c., Nat-m., Sil., Marum-v., Zinc.
— over right scapula, Bary-c., Can-s., Caust., Iod., Lauroc., Senega., Sul., Ver.
— running pain in left scapula, Sil.
— pressure in spine extending across dorsal region, and in scapulæ like that produced by sewing, Sepia.
— first a pressure then a burning in right scapula, Lyco.
— and pricking in left scapula, Fluor-ac.
— and pressure on lower end of right scapula, frequently recurring, disappearing by motion, Nat-c.
— and heat in dorsal region mostly between scapula while sitting *reading* at night, *Helon.*
— in *right* scapula, *Carbo-v.*, *Lyco.*, Lachn.
— and soreness between scapulæ, Phos-ac.
— in lower portion of scapulæ and *spine*, Pallad.
— in a small spot on left side of lumbar vertebræ and at same time in lower end of left scapula, worse from arising from a seat, better when walking, Bary-c.
— and shooting violently on upper part of scapulæ, passing off only for a short time by rubbing, Stann.
— on skin of right scapula, Zinc.
— with sticking in middle of right scapula, Lauroc.
— as from stitches in scapulæ, *Nux-v.*
— stitch on outer border of right scapula, Bary-c.
— stitch on top of right shoulder, Stann.
— stitch frequently on left shoulder, Graph.
— stitches, fine, under right scapula, extending toward ribs, Asaf.
— and throbbing between scapulæ, *Phos.*
— under left scapula with pain through shoulder, *Cund.*
— between scapulæ from vomiting, *Rhus.*
— in muscles about lower margins of scapulæ in women who follow sedentary employment, often over a small space,

SCAPULÆ.

greatly worse by long-continued needle-work or writing, *Ran-b.*

**Burning** in upper end of left scapula, somewhat better by rubbing, Alumina.

— in upper part of right scapula, Bary-c.

— in left scapula, as if hot water were poured over it, Nat-m.

**Changing,** pain beneath right scapula, changing to a stitch upon respiration, Cup.

**Chest,** pain between scapulæ and on chest, Lach.

— constant pain under lower angle of right scapula, extending to chest or stomach, causing nausea and vomiting, *Chelid.*

— cutting, crampy pain through left side to chest and scapulæ, *Nat-m.*

